# Transcriptional Profiling of the *Candida albicans* Response to the DNA Damage Agent Methyl Methanesulfonate

**DOI:** 10.3390/ijms23147555

**Published:** 2022-07-07

**Authors:** Yuting Feng, Yan Zhang, Jie Li, Raha Parvizi Omran, Malcolm Whiteway, Jinrong Feng

**Affiliations:** 1 Department of Pathogen Biology, School of Medicine, Nantong University, Nantong 226007, China; fyt18036440006@163.com (Y.F.); zy720189@163.com (Y.Z.); lee10052022@163.com (J.L.); 2 Biology Department, Concordia University, Montreal, QC H4B 1R6, Canada; raha.parvizi.o@gmail.com (R.P.O.); malcolm.whiteway@concordia.ca (M.W.)

**Keywords:** DNA damage response, methyl methanesulfonate, RNA-seq, Rad53, *Candida albicans*

## Abstract

The infection of a mammalian host by the pathogenic fungus *Candida albicans* involves fungal resistance to reactive oxygen species (ROS)—induced DNA damage stress generated by the defending macrophages or neutrophils. Thus, the DNA damage response in *C. albicans* may contribute to its pathogenicity. Uncovering the transcriptional changes triggered by the DNA damage—inducing agent MMS in many model organisms has enhanced the understanding of their DNA damage response processes. However, the transcriptional regulation triggered by MMS remains unclear in *C. albicans*. Here, we explored the global transcription profile in response to MMS in *C. albicans* and identified 306 defined genes whose transcription was significantly affected by MMS. Only a few MMS-responsive genes, such as *MGT1*, *DDR48*, *MAG1,* and *RAD7*, showed potential roles in DNA repair. GO term analysis revealed that a large number of induced genes were involved in antioxidation responses, and some downregulated genes were involved in nucleosome packing and IMP biosynthesis. Nevertheless, phenotypic assays revealed that MMS-induced antioxidation gene *CAP1* and glutathione metabolism genes *GST2* and *GST3* showed no direct roles in MMS resistance. Furthermore, the altered transcription of several MMS—responsive genes exhibited *RAD53*—related regulation. Intriguingly, the transcription profile in response to MMS in *C. albicans* shared a limited similarity with the pattern in *S. cerevisiae*, including *COX17*, *PRI2*, and *MGT1*. Overall, *C. albicans* cells exhibit global transcriptional changes to the DNA damage agent MMS; these findings improve our understanding of this pathogen’s DNA damage response pathways.

## 1. Introduction

The integrity and stability of the genome are critical for cells to transmit genetic information faithfully from one generation to the next [1]. However, several intrinsic and extrinsic factors, including cell metabolism, replication stress, radiation, and viral infection, may stress genomic DNA, introducing a variety of DNA lesions [2,3]. In particular, reactive oxygen species (ROS) and other active molecules generated by host immune cells cause oxidative DNA damage stress on pathogenic microorganisms [4,5]. To defend against such damage-inducing events, DNA damage response (DDR) pathways are employed for cells to sense and fix damaged DNA, allowing successful colonization in hosts [6,7,8].

The DNA damage response, consisting of pathways for the DNA damage checkpoint, chromatin remodeling, DNA repair, and DNA-damage tolerance, has been extensively studied in the budding yeast *Saccharomyces cerevisiae*. The DNA damage checkpoint plays a central role in coordinating cell cycle arrest, replication fork stalling, and transcriptional changes to facilitate the repair process. The sensor kinase Mec1 mediates a classical checkpoint activation pathway, where its activation phosphorylates, with the participation of adaptors Rad9 or Mrc1, the downstream effector kinase Rad53. The activated Rad53 further regulates the transcription of DNA damage response genes and dNTP pools directly or in a Dun1-dependent manner [4]. Subsequently, various DNA damage repair systems, including homologous recombination (HR), non-homologous end-joining (NHEJ), base excision repair (BER), nucleotide excision repair (NER), DNA postreplication repair (PRR), or DNA mismatch repair (MMR), are recruited to various DNA lesions to repair damaged DNA [9]. To ensure effective repair, cells need to induce several cellular responses, such as chromatin remodeling. Uncovering the cellular response to different DNA damage stresses has increased the understanding of the DNA damage repair machinery in *S. cerevisiae*.

Methyl methanesulfonate (MMS) is an extensively used DNA damage-inducing agent that causes alkylating damage to DNA, leading to single-strand breaks (SSBs), which may further cause double-strand breaks (DSBs) during S-phase [10]. Several studies have identified the transcription profile of *S. cerevisiae* cells to MMS stress and revealed the upregulation of DNA damage repair genes upon MMS exposure [10,11,12,13,14,15,16,17]. For example, according to several independent studies, *DOA1*, encoding a double-strand break repair protein, and *HSM3*, encoding a mismatch repair protein, are upregulated upon MMS treatment, suggesting their important roles in MMS-induced DNA damage response [13,14,18,19,20]. Similarly, the nucleotide excision repair genes *RAD16, RAD7,* and *RAD26* are also upregulated in response to MMS, suggesting that multiple repair pathways are involved in fixing MMS-induced DNA lesions in *S. cerevisiae* [13,14]. Consistent with the expression results, phenotypic assays reveal that mutation of *DOA1* or *RAD26* confers MMS sensitivity [21,22]. Emerging studies also indicate that several DDR-unrelated genes show upregulated transcription in response to MMS in *S. cerevisiae*. In particular, genes involved in nitrogen and sulfur metabolism, carbohydrate metabolism/fermentation, and mRNA processing are significantly upregulated [23]. One explanation could be that MMS acts not only on DNA but also on other cellular components such as proteins and lipids; thus, generating multiple cellular stresses. The upregulation of protein metabolism genes, such as *UBC8* and *PRE3*, may serve to replace alkylated proteins [13,24,25].

MMS exposure also suppresses the transcription of a large number of genes in *S. cerevisiae*. According to current data, most downregulated genes are involved in nucleotide and RNA synthesis, ribosomal protein synthesis, and assembly [10,17]. Several ribosomal protein-encoding genes, including *RPS10B*, *RPS22B*, *RPL39*, and *RPL27A,* are significantly repressed in response to MMS in *S. cerevisiae* [13,14]. The possibility could be that the modest reduction in ribosomal protein allows energy to be reshuffled for the increased expression of genes involved in the protective response [10]. In addition, some DDR unrelated genes such as *DBP2* and *LSM7* are repressed by MMS stress. Deleting *LSM7* increases the resistance to MMS, suggesting the potential negative roles of these repressed genes in MMS response [26].

In the pathogenic yeast *Candida albicans*, several DNA damage response genes have been identified that show similar roles to their orthologs in *S. cerevisiae* [4]. Moreover, DNA damage repair-related genes also contribute to the pathogenicity of *C. albicans* cells. Losing the HR element Rad52 and the NHEJ ligase Lig4 impair the pathogenicity of *C. albicans* cells [27,28]. As well, the protein phosphatase Pph3 contributes to the dephosphorylation of checkpoint Rad53 and plays a negative role in virulence, probably depending on its role in checkpoint-related morphology regulation [29,30]. However, current studies reveal that the DNA damage response in *Candida* species is not entirely the same as that in *S. cerevisiae*, although they have a close genetic framework. For instance, checkpoint kinase *RAD53* is essential in *S. cerevisiae*, but its ortholog in *C. albicans* is nonessential. The protein phosphatase 4 (PP4) complex in *C. albicans* regulates the phosphorylation of Rad53 and γH2A in a different manner from that in *S. cerevisiae* [29]. Moreover, Rad53 in *Candida glabrata* is not phosphorylated when exposed to the DNA damage inducing agent MMS, and the transcriptional profile of DNA damage response genes is significantly different from the pattern in *S. cerevisiae* [31]. Therefore, applying the identified DNA damage response pathways in *S. cerevisiae* to study the DDR in *C. albicans* and other *Candida* species may be unreliable. 

The transcriptional profile to MMS has been established in several model pathogenic fungi, such as *C. glabrata* [31] and *Fusarium oxysporum* [32], and the signaling patterns help explain their pathogenicity and drug resistance. In contrast, the lack of a global transcription profile of *C. albicans* cells in response to MMS limits the understanding of its specific DNA damage response and pathogenicity. To this end, we established the transcriptional consequences of *C. albicans* cells in response to MMS by performing RNA-sequencing (RNA-seq) assays. We find that DNA damage response genes, antioxidant genes, and nucleosome packing genes are influenced by MMS in *C. albicans*. Moreover, *RAD53* plays an important role in regulating MMS-responsive genes. However, the transcriptional profile in response to MMS in *C. albicans* shows a limited overlap with the pattern in *S. cerevisiae*. Our study thus sheds important light on understanding the specific DNA damage response in the pathogenic fungus *C. albicans*.

## 2. Results

### 2.1. MMS Treatment Inhibits the Separation and Growth of C. albicans Cells 

**Figure 1:**
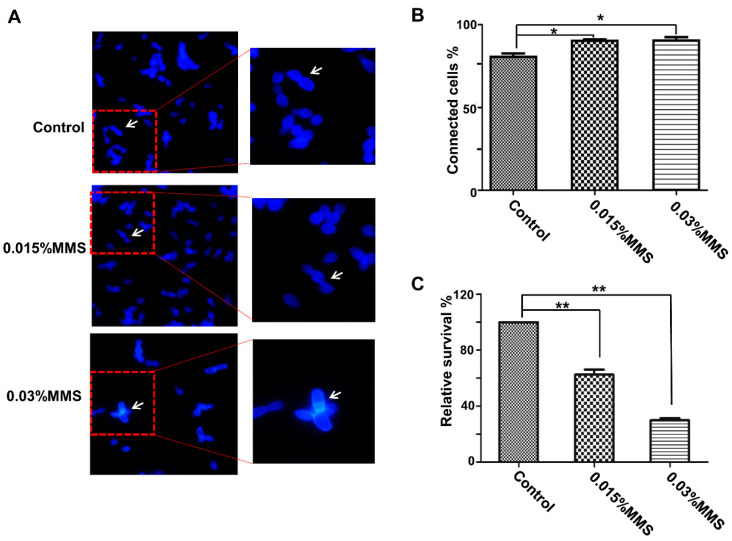
Effect of MMS on the growth of *C. albicans* cells. (**A**,**B**) Morphology of *C. albicans* cells with the MMS treatment. The log phase WT (SN148) cells were treated with 0.015% or 0.03% MMS for 90 min, before being stained with CFW (×400). The single and connected cells in panel (**A**) were counted, and the ratio of connected cells was listed. (**C**) Survival analysis of *C. albicans* cells with the MMS treatment. The log phase WT (SN148) cells were treated with 0.015% or 0.03% MMS for 90 min before being spread on YPD plates. The CFUs surviving from MMS treatment were counted and compared with the non-treated group (n = 3). The connected cells were indicated by arrows. * represents *p* < 0.05 and ** represents *p* < 0.01.

MMS is considered as a genotoxic agent and introduces lesions on DNA. Based on our previous studies, long-time treatment of MMS, such as 4 or 6 h, leads to elongation of *C. albicans* cells, which will provide an independent signal that may confuse our understanding of the response to MMS [29]. Furthermore, the accumulation of damaged DNA induces the phosphorylation of checkpoint Rad53. According to our previous studies [29], short-time treatment of MMS (less than 60 min) does not lead to Rad53 being fully phosphorylated, and the treatment of MMS for 90 min is sufficient to cause significant DNA damage inside cells which induces the DNA damage response to MMS. To assess the effect of MMS on *C. albicans* cells, we used a low-concentration (0.015%) and a high-concentration (0.03%) MMS to treat *C. albicans* for 90 min and checked the morphology and survivability. The MMS treatment significantly impaired the cell separation (Figure 1A,B); the 0.03% MMS group contained 91.3% connected cells, and the 0.015% MMS-treated group had 91% connected cells, whereas the non-treated group only had 81.4% connected cells, suggesting a defect in cell separation. In addition, MMS treatment also inhibits the growth of *C. albicans* cells under MMS stress conditions; the treatment with 0.03% MMS decreased the survival rate to 29.8%, compared to the non-treated group. Moreover, in the non-treated group, there were 1.26% dead cells, whereas there were 2.25% dead cells in the 0.015% MMS group and 3.8% dead cells in the 0.03% MMS group. Overall, the MMS treatment significantly inhibited the growth of *C. albicans* cells under MMS stress conditions.

### 2.2. Transcriptional Profiling of the Response to MMS

**Figure 2:**
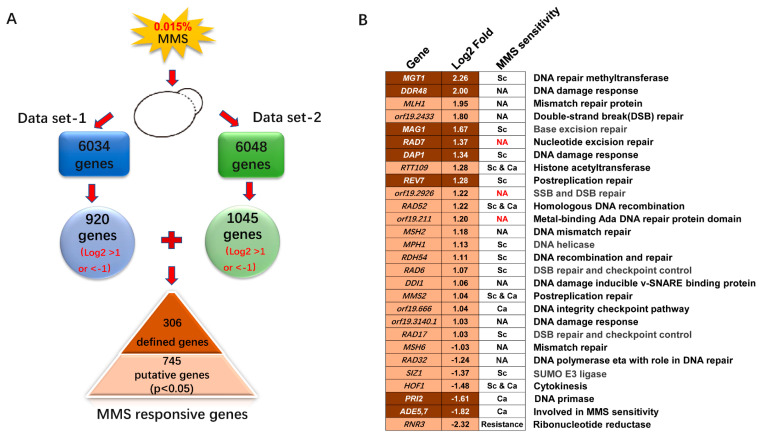
DNA damage response genes affected by MMS in *C. albicans*. (**A**) Overview of pooling transcriptional altered genes induced by MMS. Two RNA-seq assays were run independently with similar MMS treatment. The genes were pooled out from two independent RNA-seq assays by log2 fold cut-off of 1. The 306 common genes repeated in two RNA-seq assays were considered as defined MMS-responsive genes. The remaining genes with log2 fold change higher than 1 and *p* value less than 0.05 were considered as putative MMS-responsive genes in *C. albicans*. (**B**) Potential DNA damage response genes affected by MMS treatment. The genes with predicted roles in DNA damage response were selected. The log2 fold change for each gene was shown. The gene with dark background came from the defined MMS-responsive gene group, and the gene with light background came from the putative MMS-responsive gene group. The phenotype in response to MMS of mutant strains of mentioned genes was shown. Sc: *S. cerevisiae*; Ca: *C. albicans*.

The 0.015% MMS treatment significantly inhibits the viability of *C. albicans* cells and initiates the DNA damage checkpoints, according to a previous study [33]. We used this concentration to treat *C. albicans* cells for further transcriptome sequencing. Overall, 6034 or 6048 transcripts were detected in two independent RNA-seq assays, covering 94.0% or 94.2% of the ORFs in *C. albicans,* respectively (Figure 2A). 

By using a log2 fold cut-off of 1.0, we selected 920 genes in data set 1 (Table S1) and 1045 genes in data set 2 (Table S2). Among the two data sets, we found that 306 genes, including 215 upregulated and 91 downregulated genes, showed consistent transcription, suggesting defined MMS-inducible transcription. The log2 fold change values from two independent assays were averaged and shown in Table S3. In addition, the remaining genes from the two sets of data were combined and selected by a *p* value less than 0.05, generating 420 upregulated and 325 downregulated genes, that could be putative MMS-responsive genes (Table S4). Overall, 1051 genes in *C. albicans* showed significant transcriptional changes to the specific MMS treatment (0.015% MMS for 90 min) according to our results (Figure 2A).

### 2.3. DNA Damage Response Genes Induced by MMS in C. albicans

MMS is an alkylating agent and acts as a mutagen by adding methyl groups to DNA at 7-guanine, 3-guanine, and 3-adenine, leading to DNA lesions. Therefore, we anticipated identifying specific DNA damage response genes induced by MMS in *C. albicans*. We checked all the defined MMS-responsive genes and selected genes according to their functions in *C. albicans*, or the functions of their orthologs in *S. cerevisiae*. We analyzed the defined and putative sets of genes for genes involved in the DNA damage response. Seven genes with clear roles in the DNA damage response were identified in the defined group, and 19 genes were identified in the putative group (Figure 2B). In general, 26 genes with predicted roles in DNA damage response showed altered transcription to MMS, which only covers 2.47% of MMS-responsive genes (Figure 2B).

The MMS treatment may induce SSBs on DNA, generating toxic DSBs during S-phase [34]. Repairing DSBs mainly relies on the homologous recombination (HR) and non-homologous end joining (NHEJ) pathways. As expected, we found that *orf19.2433, orf19.2926/PSO2, RAD52*, *RDH54*, *RAD6*, and *RAD17* with predicted roles in DSB repair or DNA recombination were clearly upregulated. As well, histone acetyltransferase *RTT109* was upregulated; its ortholog in *S. cerevisiae* functions in the NHEJ pathway through interaction with ScVps75 [35]. DNA helicases have major roles in genome maintenance by unwinding structured nucleic acids and thus are involved in HR, NER, and PPR pathways [4,36]. Here, a putative 3’-5’ DNA helicase *MPH1* was induced with MMS treatment in *C. albicans*. Overall, lack of *RAD52*, *RTT109*, *RDH54*, *RAD6*, *RAD17,* and *MPH1* confers MMS sensitivity either in *C. albicans* or *S. cerevisiae*, suggesting essential involvements in treating MMS-induced DNA damage stress [21,37,38,39,40,41,42]. The *RAD7*, *orf19.1926,* and *orf19.211* mutants are found in the GRACE library but were not reported as MMS-sensitive strains, suggesting no involvement in MMS resistance of these genes [33]. In general, most of the DNA damage response genes affected by MMS showed a requirement in resistance to MMS either in *S. cerevisiae*, *C. albicans*, or both.

Moreover, DNA damage repair genes that belong to other repair pathways also showed altered transcription in response to MMS. The nucleotide excision repair (NER) gene *RAD7*, which is mainly involved in repairing ultraviolet (UV) -induced DNA damage, was upregulated. As well, base excision repair (BER) gene *MAG1*, postreplication repair genes *REV7* and *MMS2* were all upregulated. However, mismatch repair genes *MLH1* and *MSH2* were induced while the *MSH6* gene was repressed. DNA polymerase *RAD32* was suppressed by MMS, which may contribute to the fork stalling upon MMS stress. In addition, the putative DNA repair methyltransferase *MGT1,* whose ortholog protects against DNA alkylation damage in *S. cerevisiae*, showed the highest upregulation and probably has a critical role in withstanding MMS stress. Phenotypic data in Saccharomyces Genome Database (SGD) indicates that deleting *MAG1*, *REV7*, *MMS2*, and *MGT1* confers MMS sensitivity, suggesting critical roles in response to MMS [21,43,44]. The transcription of some DNA damage response genes, including *DDR48*, *DAP1,* and *orf19.3140.1,* were upregulated in response to MMS. Furthermore, *HOF1* and *ADE5,7*, with the contribution to MMS resistance, showed an apparent downregulation in response to MMS; this result is consistent with the reported decreased protein level of Hof1 in response to MMS [33]. Furthermore, *RNR3* was downregulated upon MMS exposure, and deleting *RNR3* resulted in increased resistance to MMS, suggesting a negative role in response to MMS.

In addition, we observed that several checkpoint kinases *MEC1*, *RAD9*, *RAD53*, *DUN1,* and many other DDR genes, did not show significantly altered transcription, which is consistent with a previous finding that checkpoints mainly rely on posttranslational regulation in *S. cerevisiae* [45]. *DUN1* was clearly upregulated by MMS treatment in *S. cerevisiae* [11], and in *C. albicans* showed negligible change in response to MMS, according to our two independent RNA-seq assays. In general, limited DNA damage response genes showed altered transcription to MMS, and most of them had moderate upregulation (log2 fold change less than 2, except *MGT1* and *DDR48*) in response to MMS.

### 2.4. Evaluation of MMS-Responsive Genes

As shown above, few MMS inducible genes were involved in direct DNA damage response, but most of them showed no direct linkage to DDR. To identify the patterns of differential gene transcription, GO analysis was performed using the FungiFun online tool (https://elbe.hki-jena.de/fungifun/) (accessed on 12 March 2022) [46]. The changed genes were categorized according to the biological processes they were involved. In general, several GO terms, including oxidation–reduction process, cellular response to oxidative stress, ‘de novo’ IMP biosynthetic process, and nucleosome assembly, were enriched (Table S5). 

#### 2.4.1. Oxidative Stress-Related Genes

**Figure 3:**
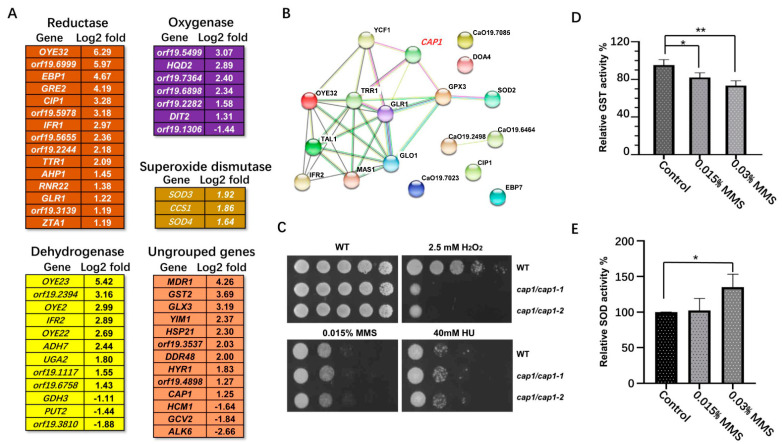
Oxidative stress genes affected by MMS in *C. albicans*. (**A**) Summary of typical oxidation response genes affected by MMS. The log2 fold change for each gene from the defined MMS-responsive group was listed behind the gene name. (**B**) The Cap1 related genes are induced by MMS. The mentioned genes were analyzed by the STRING platform. (**C**) The phenotypic assay of the *CAP1* deletion strain to H_2_O_2_ and genotoxic stresses. (D&E) The total GST activity (**D**) and the total SOD activity (**E**) affected by MMS treatment. The WT strain (SN148, n = 3) was treated with 0.015% or 0.03% MMS for 90 min before being harvested for activity assay. The relative activity was compared to the enzyme activity in the untreated group, where its activity was normalized as 1. * represents *p* < 0.05 and ** represents *p* < 0.01.

A large number of changed genes were assigned to oxidative response processes. Among these, 39 genes were assigned to oxidation-reduction process (GO:0055114), and 14 genes were assigned to the cellular response to oxidative stress (GO:0034599). Overall, 51 different oxidative genes from the defined group showed altered transcription in response to MMS (Figure 3A).

We found 15 reductase encoding genes, including *OYE32*, *orf19.6999,* *EBP1*, *GRE2*, *CIP1, orf19.5978*, *IFR1*, *orf19.5655*, *orf19.2244*, *TTR1*, *AHP1*, *RNR22, GLR1, orf19.3139,* and *ZTA1* that all showed increased transcription. In particular, the transcription of *OYE32,* encoding a NAD(P)H oxidoreductase, showed the highest upregulation, with a log2 fold of 6.29. In addition, 9 dehydrogenase encoding genes *OYE23, orf19.6999*, *OYE2, IFR2, OYE22, ADH7, UGA2, orf19.1117*, and *orf19.66758* showed significantly increased transcription in response to MMS, while only *GDH3, PUT2,* and *orf19.3810* were moderately downregulated. Oxygenases *orf19.5499, HQD2, orf19.7364, orf19.6898, orf19.6898, orf19.2282,* and *DIT2* were upregulated with MMS treatment; only a predicted 2-oxoglutarate-dependent dioxygenase *orf19.1306* was downregulated. Furthermore, the plasma membrane MDR/MFS multidrug efflux pump *MDR1*, the glutathione S transferase *GST2,* and the glutathione-independent glyoxalase *GLX3* showed dramatically increased transcription. However, *ALK6*, encoding a putative cytochrome P-450 of N-alkane-induced detoxification, was significantly downregulated. In general, the antioxidation response genes showed general upregulation with MMS exposure.

Cap1 is a bZip transcription factor involved in oxidative stress response in *C. albicans* [47]. *CAP1*- and 5-regulated genes, including *OYE32*, *IFR2*, *CIP1*, *ADH7*, and *GLR1*, were all upregulated in response to MMS. In agreement, 14 Cap1-regulated/related genes were found from the putative MMS-responsive gene group, and these Cap1-regulated/related genes all showed clear upregulation. The STRING analysis showed that most of these Cap1-regulated/related genes have functional correlation (Figure 3B), suggesting a central role of *CAP1* in coordinating the antioxidation response to MMS. As well, ScYAP1, the ortholog of *CAP1* in *S. cerevisiae*, and its targets show stable upregulation in response to MMS, suggesting a conserved activation of transcription factor Cap1/Yap1 during this process [11]. To better illuminate the function of *CAP1* in response to MMS, we deleted the *CAP1* gene and tested for MMS sensitivity. Consistent with previous studies [48], *CAP1* deletion resulted in strong sensitivity to H_2_O_2_, but generated no clear defect in response to MMS and HU (Figure 3C). 

The significant change in oxidative-stress-related genes implied that MMS impaired the cellular redox equilibrium. To confirm this idea, we used 0.015% and 0.03% MMS to treat the log-phase cells and tested the total GST activity and SOD activity. With MMS treatment, the total GST activity decreased significantly, compared with the untreated group; 0.015% MMS treatment decreased the GST activity level to 81.9%, while 0.03% MMS treatment decreased the GST activity level to 73.3% (Figure 3C). However, the total SOD activity increased with MMS treatment; in particular, the high concentration of MMS increased the total SOD activity level to 135.1%, showing a significant difference from the untreated group (Figure 3D).

#### 2.4.2. ‘De Novo’ IMP Biosynthetic Process

**Figure 4:**
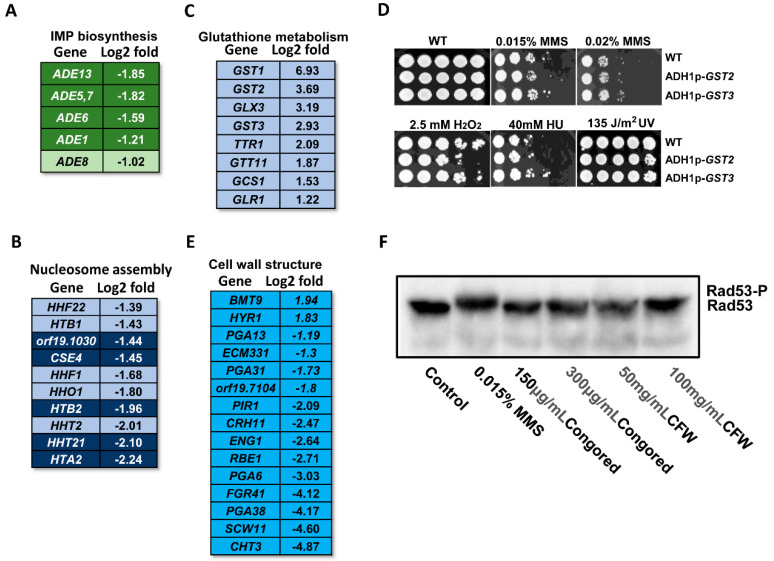
Summary of IMP biosynthetic process, nucleosome assembly, glutathione metabolism, and cell wall structure genes affected by MMS in *C. albicans*. (**A**) IMP biosynthetic process related genes. (**B**) Nucleosome assembly related genes. (**C**) Glutathione metabolism genes. (**D**) Phenotypic assay by increasing the transcription of *GST2* and *GST3*. (**E**) Cell wall structure genes. (**F**) Anti-HA Western blot of WT with Rad53-HA that was incubated with MMS, Congo Red, or CFW for 90 min. In panels (**A**,**B**), the genes with a dark background came from the defined MMS-responsive gene group, and the genes with a light background came from the putative MMS-responsive gene group. In panels (**C**,**E**), all the genes came from the defined MMS-responsive gene group. The value of log2 fold for each gene was listed behind the gene name.

The ‘*de novo*’ inosine 5′-monophosphate (IMP) biosynthetic process represents the chemical reactions and pathways resulting in the formation of IMP, inosine monophosphate, by the stepwise assembly of a purine ring on ribose 5-phosphate. Here, phosphoribosylamine-glycine ligase *ADE5,7*, phosphoribosyl aminoimidazole succinocarboxamide synthetase *ADE1*, 5-Phosphoribosylformyl glycinamidine synthetase *ADE6*, and adenylosuccinate lyase *ADE13* from the defined MMS-responsive gene group were all downregulated (Figure 4A). In addition, we found that phosphoribosylglycinamide formyl-transferase *ADE8* from the putative MMS-responsive gene set was also downregulated. Therefore, the extensive downregulation of ADE genes suggests a slowdown of purine synthesis. 

#### 2.4.3. Nucleosome Assembly

Nucleosome assembly is coupled to DNA replication and repair. Here, we found that several nucleosome assembly genes were downregulated (Figure 4B). Histone synthesis genes *CSE4*, *HTB2*, *HHF21*, and *HTA2* were downregulated in response to MMS. As well, a putative peptidyl-prolyl cis-trans isomerase *orf19.1030* was also downregulated. Moreover, downregulated histone genes *HHF22*, *HTB1*, *HHF1*, *HHO1*, and *HHT2* from the putative MMS-responsive gene set were enriched for the nucleosome assembly process. This result is consistent with the observation in *S. cerevisiae* that histone encoding genes *HTA1*, *HTA1*, and *HTB2* are significantly repressed by MMS treatment [10], suggesting a potential conservative reaction of the nucleosome assembly process to MMS-induced DNA damage stress.

#### 2.4.4. Glutathione Metabolism 

Besides the GO enriched genes, the ungrouped genes also showed functional correlation. Among the upregulated genes, we found that a putative glutathione S-transferase *GST1* showed the highest upregulation, with a log2 fold change of 6.93. Similarly, glutathione S-transferase encoding genes *GST2*, *GST3*, and *GTT11* showed significant upregulation, with log2 fold changes greater than 2 (Figure 4C). As well, some other glutathione metabolism genes *GLX3*, *TTR1*, *GCS1*, and *GLR1* were all upregulated. 

Glutathione is a ubiquitous thiol-containing tripeptide implicated in redox and detoxification [49]. Previous studies in mammalian systems suggest that MMS reduces cellular glutathione pools, leading to perturbation of the cellular redox [11]. The extensive upregulation of glutathione metabolism genes could be a side effect caused by MMS. To test this idea, we increased the transcription of *GST2* or *GST3* by introducing ADH1p-driven ectopic *GST2* or *GST3* into a wild-type strain of *C. albicans*. Increasing the expression of *GST2*, but not *GST3* decreased the resistance to H_2_O_2_. Since deletion of *GST2* also results in the sensitivity to H_2_O_2_, *GST2* could play a critical role in resistance to oxidation stress [50]. However, over-expressing *GST2* and *GST3* did not increase or decrease the resistance to MMS and the other genotoxic stresses HU or UV light, yet these genes are highly induced upon MMS exposure (Figure 4D). 

#### 2.4.5. Cell Wall Genes

DNA damage events occur inside the cell nucleus; thus, the transcriptional change in the cell wall and extracellular genes is somewhat unexpected. However, we noticed that several cell-wall-related genes showed significant downregulation. The major chitinase *CHT3* showed the most downregulation, with a log2 value of 4.87. In addition, GPI-anchored protein encoding genes *PGA38*, *FGR41*, *PGA6*, *CRH11*, *PGA31*, *ECM331*, and *PGA13* were all downregulated, while GPI-anchored hyphal cell wall gene *HYR1* was moderately upregulated. Moreover, cell wall genes *SCW11*, *RBE1*, *ENG1*, *PIR1*, and *orf19.7104* were all significantly downregulated; only beta-mannosyltransferase *BMT9* was upregulated (Figure 4E). Overall, MMS exposure induces an extensive downregulation of cell wall genes. 

The extensive alteration of cell wall structure genes raises the possible correlation between cell wall integrity and DNA damage response. Upon DNA damage stress, the checkpoint kinase Rad53 is activated and shows a slower migration rate on SDS-PAGE gels, indicating a phosphorylated form of Rad53. Here, we used cell wall stressing agents, Congo Red and CFW, to treat wild-type cells and checked the phosphorylation of Rad53. Neither Congo Red nor CFW could induce the phosphorylation of Rad53, which may partially preclude the potential correlation between cell wall integrity and DNA damage response (Figure 4F). 

### 2.5. Rad53 Plays a Critical Role in Coordinating MMS-Induced Transcription

**Figure 5:**
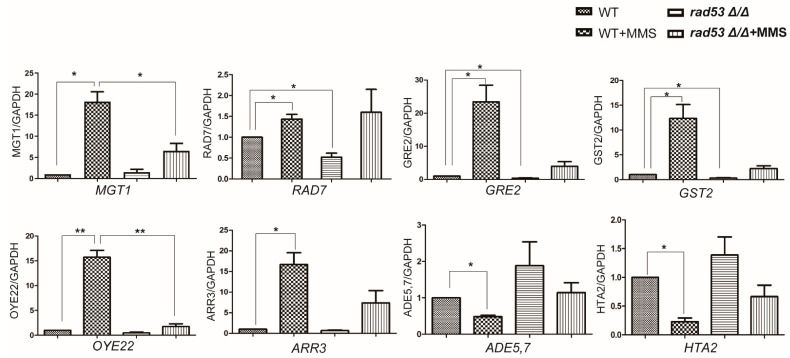
Confirmation of gene transcription by qRT-PCR. The WT strain (SN148) and the *RAD53* deletion strain were treated with 0.015% MMS for 90 min before being harvested for RNA extraction. The qRT-PCR assay for each strain contained at least 3 biological replicates. The difference between each group was compared using paired *t* test with GraphPad Prism 8 software. * represents *p* < 0.05 and ** represents *p* < 0.01.

DNA damage checkpoints play critical roles in DNA damage response through coordinating cell cycle arrest and DNA damage repair. Rad53 is a primary DNA damage checkpoint kinase and is activated in response to MMS in both *S. cerevisiae* and *C. albicans*. Therefore, we asked the question whether the changed transcription of MMS-responsive genes is regulated by Rad53. First, we checked the transcription of DDR genes after deleting *RAD53* (Figure 5). The transcription of *MGT1* and *RAD7* showed clear upregulation, while *ADE5,7* was downregulated in response to MMS, which is consistent with our RNA-seq data. After deleting *RAD53*, the transcription of *MGT1* and *RAD7* was impaired; *MGT1* was slightly upregulated by MMS stress in the *RAD53* deletion strain, but the transcription level is significantly lower than the level in the wild-type strain. For *RAD7*, its transcription is much lower than the level in wild-type strain in the no MMS stress condition; but in the MMS stress condition, the level of *RAD7* in the *RAD53* deletion group is similar to the level in the WT group. Moreover, *ADE5,7* was downregulated in response to MMS in wild-type strain, but its downregulation was blocked by deleting *RAD53*; the transcription of *ADE5,7* remained the level in wild-type strain, either in normal or DNA damaging conditions. These observations suggest a critical role of *RAD53* in regulating the transcription of DNA damage response genes. 

We also checked the potential transcriptional regulation of Rad53 on several DDR unrelated genes (Figure 5). The upregulation of *GRE2*, *GST2, OYE22, ARR3,* and the downregulation of *HTA2* in response to MMS was consistent with the RNA-seq data. The transcription level of *GRE2* and *GST2* decreased significantly (37.8% for *GRE2* and 31% for *GST2*) by deleting *RAD53* in no-stress conditions; in the MMS stress situation, they showed a reduced transcription (16.8% for *GRE2* and 18.1% for *GST2*)) but with no significant difference compared to the level in wild-type strain. The transcription of *OYE22* showed a clear downregulation by deleting *RAD53* in the MMS stress condition. As well, the downregulation of histone encoding gene *HTA2* in response to MMS was blocked by deleting *RAD53*, either in normal or MMS stress conditions. However, the transcription of *ARR3,* encoding an arsenite transporter, was not significantly affected by deleting *RAD53*. Overall, Rad53 appears to regulate a broad range of target genes in MMS stress conditions.

### 2.6. Differential Transcriptome to MMS in C. albicans and S. cerevisiae

**Figure 6:**
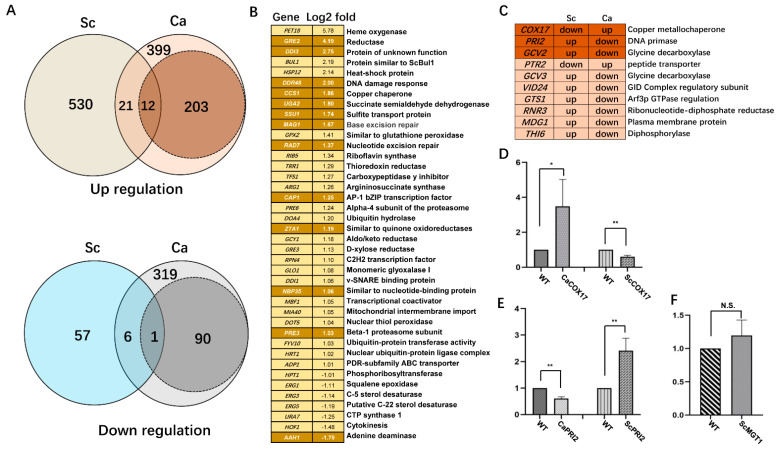
Comparison of the transcriptional profile to MMS in *S. cerevisiae* and *C. albicans*. The MMS-responsive transcriptional data was collected from published papers with fold changes over 2 fold. These data sets were checked and combined. The genes reported twice or more were considered as core MMS-responsive genes in *S. cerevisiae* and were used to check the similarity with the transcriptional profile to MMS in *C. albicans*. Both the defined MMS-responsive gene set and the putative MMS-responsive gene group were compared with the core MMS-responsive genes in *S. cerevisiae.* The similarity between different data sets was compared (**A**), and the common genes were listed in panel (**B**). The gene name with a dark background came from the defined MMS-responsive gene group, and the gene with a light background came from the putative MMS-responsive gene group. Sc: *S. cerevisiae*; Ca: *C. albicans*. (**C**) The genes with opposite transcription responses to MMS in *S. cerevisiae* and *C. albicans* were shown. (**D**–**F**) qRT-PCR assay of *COX17*, *PRI2,* and *MGT1* in *S. cerevisiae* and *C. albicans*. The WT strains of *S. cerevisiae* (BY4741) and *C. albicans* (SN148) were treated with 0.015% MMS for 90 min before being harvested for RNA extraction. The qRT-PCR assay for each strain contained at least 3 biological replicates. The difference between each group was compared using paired *t* test with GraphPad Prism 8 software. * represents *p* < 0.05 and ** represents *p* < 0.01.

*S. cerevisiae* and *C. albicans* have close genetic frameworks, and thus a similar transcriptional pattern in response to different stress conditions, including MMS, may be expected. Several independent studies have provided transcriptional profiles of yeast cells responding to MMS, using MMS doses ranging from 0.01% to 0.1% with treated times ranging from 90 to 120 min [10,11,12,13,14,15,16,17]. The data from those published papers with similar treatments were combined, and the genes with consistent transcription repeated in at least two of these data sets were considered as the core MMS-responsive genes in *S. cerevisiae*. In total, we extracted 563 upregulated and 64 downregulated genes (Table S6). Strikingly, the transcriptional data in *C. albicans* showed only a limited consistency with the core MMS-responsive transcriptional profile in *S. cerevisiae*; only 12 induced genes, including DNA damage response or repair genes *MAG1*, *RAD7, DAP1,* and *DDR48,* were upregulated by MMS stress both in *S. cerevisiae* and *C. albicans* (Figure 6). For the repressed group, only the adenine deaminase gene *AAH1* was downregulated by MMS stress in these two yeast species (Figure 6). Overall, a low similarity of the putative MMS-responsive gene set with the data set from *S. cerevisiae* was found; only 21 induced genes and 6 repressed genes were common in these two species. GO analysis revealed that 16 common genes, including *DDR48*, *CAP1*, and *ERG* genes, were involved in oxidation response, and 4 common genes, including *RPN4*, *PRE3*, *FYV10*, and *PRE6,* were engaged in proteasome-mediated ubiquitin-dependent protein catabolic process. The common MMS-responsive genes and biological processes suggest their conserved responding pathways to MMS in *C. albicans* and *S. cerevisiae*.

We looked into the different transcription data between these two yeast species in detail. Several MMS-responsive genes were upregulated in *C. albicans*, but downregulated in *S. cerevisiae*, including *COX17* and *PTR2*. qRT-PCR confirmed that *COX17* showed a 3.5-fold upregulation in *C. albicans*, but showed a 40% downregulation in *S. cerevisiae*, with 0.015% MMS treatment. In contrast, several MMS-responsive genes were downregulated in *C. albicans*, but upregulated in *S. cerevisiae,* including *PRI2* and *GCV2.* As well, *PRI2* was remarkedly downregulated in *C. albicans*, but showed a 2.4-fold upregulation in *S. cerevisiae* with a similar MMS treatment. Furthermore, *MGT1* was significantly upregulated in *C. albicans* with 0.015% MMS treatment but showed no significant change in *S. cerevisiae* upon a similar MMS exposure. Thus, *C. albicans* and *S. cerevisiae* do have different transcriptional responses to the DNA damage stress induced by MMS.

## 3. Discussion

**Figure 7:**
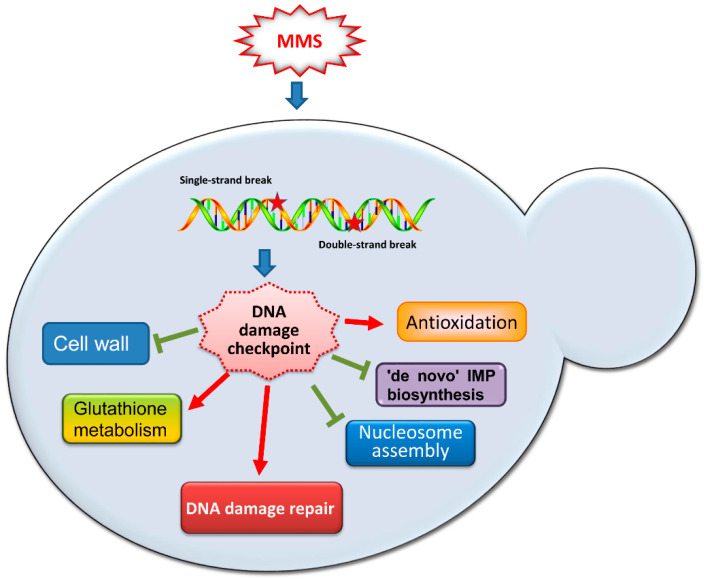
Overview of the transcriptional profile to MMS exposure in *C. albicans*. MMS functions on DNA and introduces lesions, such as SSBs or DSBs. The DNA damage checkpoints are activated to, maybe partially, regulate MMS-responsive genes. Genes involved in DNA damage repair, antioxidation process, and glutathione metabolism are induced by MMS, while nucleosome assembly ‘de novo’ IMP biosynthetic process and cell-wall-related genes are repressed.

In this study, we explored the transcription profile of *C. albicans* cells in response to the DNA-damaging agent MMS and found that *C. albicans* cells showed a dramatic transcriptional change in response to 0.015% MMS. DNA damage response and various stress-responsive processes, including antioxidation, ‘de novo’ IMP biosynthesis, and nucleosome assembly, were affected by MMS treatment (Figure 7). 

The DNA damage response pathway is engaged in withstanding intrinsic or extrinsic DNA damage stress. DNA damage checkpoints detect damaged DNA, arrest the cell cycle, and regulate repair genes to fix DNA lesions [51]. According to our data, we found no clear change in the transcription of checkpoint genes in response to MMS in *C. albicans*. Consistent with the pattern in *S. cerevisiae,* these observations indicate that the transcription of DNA damage checkpoint genes does not change dramatically upon DNA damage stress. Post-transcriptional regulation, such as the phosphorylation/dephosphorylation of Rad53, regulates the cellular function in response to DNA damage stress in *C. albicans*. Moreover, the transcription of DNA damage response genes also showed limited changes; we only found 7 out of 306 defined MMS-responsive genes with modified transcription showed potential roles in the DNA damage response. Among these potential DDR genes, *MGT1* showed the highest fold change, with log2 fold change of more than 2; the log2 values of most other DDR genes were less than 2. This finding implies that DNA damage sensing and repair genes may not rely primarily on transcriptional regulation. It could be that excessive DNA damage response proteins are toxic to normal cells. For example, overexpressing *RAD53* in *S. cerevisiae* leads to decreased resistance to UV, fluconazole, and various metal ions [52]. Moreover, overexpressing *RAD23* in *C. albicans* results in increased sensitivity to UV stress, even though deleting this gene causes severe sensitivity to UV [53]. Therefore, with proper post-transcriptional level activation, the basal level of DNA damage response genes is sufficient for correcting DNA lesions; the absence of DNA damage repair genes indeed impairs the DNA damage repair process, but excessive DNA damage repair ability may disturb normal cells. 

Genotoxic MMS induces DNA alkylation, and causes SSBs, leading to DSBs on genomic DNA. Fixing these lesions usually relies on specific repair pathways such as HR or NHEJ. Therefore, the upregulation of DSB repair genes, including typical HR genes *RAD52* and *RDH54*, is logical. Nevertheless, we also found the upregulation of several other DNA damage repair pathways involving genes such as the BER gene *MAG1*, PRR gene *REV7*, and MMR gene *MLH1.* Similar upregulation of these repair genes also happens in *S. cerevisiae* and *F. oxysporum*. According to the data set we collected from *S. cerevisiae*, nucleotide excision repair genes *RAD16* and *RAD2* are upregulated (Table S6). Similarly, the NER pathway is activated by MMS to repair bulky DNA lesions in *F. oxysporum* [32]. Thus, restoring the MMS-induced DNA damage may not only rely on a solo repair process but needs the participation of the whole repair machinery. The MMS sensitivity resulting from deleting *ScRAD2*, a subunit of nucleotide excision repair factor 3, may partially support the involvement of other repair systems in fixing MMS-induced damage. In general, MMS could be an extensive DNA damaging agent and causes dramatic lesions on genomic DNA.

Besides DNA damage repair genes, some other DNA-damage-unrelated genes showed remarkable upregulation in response to MMS stress. In particular, several oxidation-responsive genes, including *GST1*, *GST2, OYE32*, *OYE23*, *OYE22,* and *OYE2,* were significantly upregulated. According to current studies, none of these genes exhibit potential roles in DNA damage response. In independent research, deletions of the *GSTs*, even the triple *gst1 gst2 gst3* strain in *C. albicans*, showed no MMS sensitivity (unpublished data). Here, we observed that either deleting *CAP1*, or overexpressing *GST2* and *GST3* conferred no MMS sensitivity or resistance. These results may suggest that oxidation-responsive genes are not real DNA damage sensing or repairing genes; the upregulation of oxidation-responsive genes may be an indirect response to MMS. Consistent with this idea, MMS is considered as an alkylating agent and impairs redox homeostasis [54,55]. The decreased GST activity and increased SOD activity by MMS stress may support this idea. Moreover, glutathione-S-transferase genes are induced by heavy metal ion and oxidant toxicity in *S. cerevisiae* and other model organisms [56,57,58]. Thus, it is possible that the MMS treatment causes oxidative stress and further induces the transcription of GSTs and other oxidative response genes in *C. albicans*. Taken together, the cellular response induced by MMS appears to have elements that are separate from the critical function in DNA damage response, and therefore a large portion of MMS-responsive genes are not directly related to DNA damage repair.

Rad53 is a crucial DNA damage checkpoint protein and can be phosphorylated in response to MMS stress to initiate the repair process in many eukaryotic cells. Since MMS exposure induces a global transcriptional change, we asked the question whether this regulation relies on the function/activation of Rad53. Specifying the Rad53 related targets is valuable for understanding the DNA damage response in *C. albicans*. In our study, we found that the DNA repair genes *MGT1* and *RAD7* were partially regulated by *RAD53*. This finding is consistent with their important roles in DNA damage repair. However, we also found that many DNA-damage-unrelated genes, including *GRE2*, *OYE22,* and *GST2*, showed a degree of Rad53-related transcription in response to MMS. *GRE2* encodes a putative reductase, *OYE22* encodes a putative NADPH dehydrogenase, and *GST2* encodes a putative glutathione S transferase in *C. albicans*. According to current data, none of these proteins showed direct roles in DNA damage response. Moreover, the downregulation of *HTA2*, encoding a putative histone H2A, was blocked by deleting *RAD53*, implying a great degree of *RAD53*-dependent regulation. In *S. cerevisiae*, the DNA damage sensitivity and slow growth of the *rad53* mutant can be suppressed by deleting histone genes, which may support the correlation between Rad53 and histones [59]. Therefore, the current result suggests that MMS treatment activates Rad53 and induces global transcriptional responses that are not confined to DNA damage repair genes. One possibility could be the side-effect of MMS in causing oxidative stress, which further upregulates the antioxidation genes partially through the activated Rad53. Consistent with this idea, the checkpoint kinase Rad53 is activated in response to oxidative stress, suggesting the correlation between DNA damage response and antioxidation response [60]. Another possibility could be that the activation of Rad53 blocks the cell cycle progression to repair damaged DNA, and this cellular activity needs the coordination of various cellular processes. Thus, the nucleosome remodeling genes, cell wall composition genes, and ‘de novo’ IMP biosynthesis genes are induced/suppressed by MMS, and some of these genes are indirectly regulated by *RAD53*. Some studies in *S. cerevisiae* reported the ScRad53-dependent targets in response to MMS, but most of them are DNA damage repair unrelated genes such as *ABF1* and *AIM34* [61]. To better understand the transcriptional regulation by *RAD53*, RNA-seq assays based on the *RAD53* deletion strain or the *RAD53* overexpression strain can be performed in the future. In general, *RAD53* regulates a broad range of target genes in MMS stress conditions in *C. albicans*.

By comparing the transcription profiles in *C. albicans* and *S. cerevisiae,* limited genes showed consistent responses to MMS in these two yeast species. Given their close genetic framework, this extremely limited similarity during their responses to MMS exposure is unexpected. A potential explanation could be the variable data obtained through microarray or RNA-seq. In *S. cerevisiae,* Caba et al. reported 883 responsive genes showing at least 2-fold changes by 0.12% MMS (*v/v*) stress for 60 min [14], while Benton et al. found 714 genes exhibiting at least 3-fold change responding to one dose of MMS for 60 min (0.001% MMS, 0.01% MMS, or 0.1% MMS, *v/v*) [13]. In *C. albicans*, we found 306 defined MMS-responsive genes from two independent assays with a similar treatment (0.015% MMS (*v/v*) for 90 min). As reported by Caba et al. and Gasch et al. [11,14], different treatment times and doses of MMS can give rise to distinct transcription data in *S. cerevisiae*. Therefore, the specific treatment time and dose of MMS may not accurately correspond between these two species, resulting in different profiles. Furthermore, the minor change in cell status, treatment time, and dose may generate discrepant transcription profiles. Nevertheless, other reasons may also contribute to the limited similarity in MMS response. The pooling of common MMS-responsive genes from different *S. cerevisiae* studies may have missed real MMS-responsive genes. *ScRAD52*, a widely studied homologous recombination repair gene in *S. cerevisiae*, was only found to be upregulated by Benton et al. [13], and thus was not selected in the core *S. cerevisiae* data set. However, *CaRAD52* was upregulated in response to MMS in our study; deleting *RAD52* in *S. cerevisiae* and *C. albicans* both leads to remarkable MMS sensitivity, suggesting the role of *RAD52* is in fact conserved in MMS-induced DNA damage response. Finally, there are a large number of MMS-responsive genes in *S. cerevisiae* that do not have currently identified orthologs in *C. albicans*. We cannot exclude the possibility that MMS-responsive orthologs of such genes in *S. cerevisiae* could be identified in *C. albicans* in the future. Overall, the similarity of MMS-responsive genes in *S. cerevisiae* and *C. albicans* could be higher than the baseline we present based on the current data. 

Insights into the DNA damage response can be obtained by comparing the transcription data in *S. cerevisiae* and *C. albicans*, even though the similarity is at such a low level. The similar transcription of specific MMS inducible genes in these two eukaryotic cells suggests conservative roles in DNA damage response. *ScDDR48* is a DNA damage-responsive gene and plays a potential role in the production or recovery of mutants [62]; while in *C. albicans*, *CaDDR48* is significantly upregulated after UV exposure, but is depressed in the absence of *Rfx2,* a DNA binding protein involved in DNA damage response [63]. Therefore, the regulation of *DDR48* by *RFX2* implies a conservative role of *DDR48* in DNA damage response. In addition, the common downregulated gene *HOF1*, a cytokinesis regulator, was recently identified as a checkpoint-related element in *C. albicans* [33], and it was assigned in a Rad53-related pathway in response to MMS. Similarly, *HOF1* in *S. cerevisiae* is regulated by checkpoint kinase Dun1, a downstream kinase of Rad53 [64]; thus, suggesting a conservative role of Hof1 in checkpoint-related pathways. However, clear transcriptional differences responding to MMS are seen in these two yeasts. *COX17*, a copper metallochaperone, and *PRI2*, a subunit of DNA primase, showed contradictory transcription to MMS in *S. cerevisiae* and *C. albicans*. Given *COX17* and *PRI2* are not involved in DNA damage repair directly, MMS treatment may affect different cellular activities during the DNA damage stress conditions in different species. *MGT1* was not significantly changed upon MMS exposure according to our result and the data sets in *S. cerevisiae*; in contrast, *MGT1* in *C. albicans* was highly upregulated in response to MMS (Log2 fold = 2.26). The difference in *MGT1* in these two yeast species may suggest that various DNA damage repair pathways are utilized in response to DNA damage. Furthermore, the MMS-induced transcriptional diversity also happens in *C. glabrata*. The transcription profile reveals that several key protectors of genome stability are upregulated by DNA damage stress in *S. cerevisiae*, but are downregulated in *C. glabrata,* such as proliferating cell nuclear antigen (PCNA). This difference may contribute to rapidly generating genetic change and drug resistance of *C. glabrata* cells [31]. In addition, we compared the defined MMS-regulated genes with the MMS-responsive genes in *C. glabrata* that have orthologs in *S. cerevisiae* [31]. We found that 20 induced genes, including *DDR48*, *RAD7*, *CAP1*, *COX17*, and 18 repressed genes, including *HTA2*, *HTB2*, *ADE6*, show consistent transcription in these two Candida species. However, the whole MMS-induced transcriptome of the two fungal pathogens shows a low similarity. Thus, the specific DNA damage response pathways in these model organisms could be diverse according to different living environments.

Our study indicated the signals involved in response to the stress of MMS in *C. albicans*, according to the two rounds of RNA-seq assays. It is important to note that only a single stress condition was used in the current study; we may not obtain the global response to MMS in *C. albicans*. Potential time- or dose-dependent MMS-responsive genes in *C. albicans* may be omitted due to the limited MMS treatment. Therefore, to uncover the whole cellular response to MMS in *C. albicans*, expanded stress conditions can be included. In general, this study reports the first overview of the specific transcriptional regulation during the MMS-induced DNA damage response in *C. albicans* (Figure 7). MMS treatment activates DDR and increases the transcription of some DSB repair genes and NER genes in *C. albicans*. MMS stress also induces a more extensive transcriptional change that includes antioxidation, ‘*de novo*’ IMP biosynthesis, nucleosome assembly, glutathione metabolism, and cell wall organization to either facilitate the repair process or withstand the side effects caused by MMS. The potential DDR-related function and the possible regulation by DNA damage checkpoint of these MMS-responsive genes need to be investigated in the future. Our findings provide insights into the transcriptional regulation of DNA damage response in *C. albicans*.

## 4. Materials and Methods

### 4.1. Strains, Media, and Reagents

*C. albicans* strains were grown in Yeast Extract Peptone Dextrose (YPD) media (1% yeast extract, 1% peptone, and 2% glucose, Sangon Biotech, Shanghai, China) with 50 mg uridine per liter as described [65]. Wild-type strains SN148, BWP17, the *RAD53* deletion strain [33], and other mentioned strains are listed in Table S7. Methyl methanesulfonate (MMS) was purchased from Sigma (St. Louis, MO, USA). The reagents and amino acids for making media were purchased from Sangon (Shanghai, China). Solid media contained 2% agar. The primers used in this study are listed in Table S8.

### 4.2. Survival Assay of C. albicans Cells

Three single colonies of the wild-type strain SN148 were inoculated into 3 mL liquid YPD and incubated overnight at 30 °C on a 200 rpm shaker. The overnight cultures were each diluted in 10 mL fresh liquid YPD media at a ratio of 1/10 and grown for 3 h at 30 °C with shaking. Afterward, 0, 1.5, or 3 µL MMS liquid was added into the cell culture to a final concentration of 0, 0.015%, or 0.03%, and kept at 30 °C for 1.5 h. The cultures were spread on YPD plates with 10-fold serial dilutions and incubated at 30 °C for two days. The colonies on each plate were counted (CFUs/mL) and compared with the untreated group.

### 4.3. Calcofluor White (CFW) Staining

Cell samples (300 mL) from the survival assay were first fixed in 70% ethanol for 20 min and then washed twice with PBS (pH 7.4). The cells were resuspended in PBS containing 1.0 µg/mL calcofluor white for 5 min in the dark at room temperature. Afterward, they were washed twice in PBS (pH 7.4) before mounting for examination under a fluorescence microscope. Similarly, the cell samples were stained with trypan blue using a kit (E607320) from Sangon (Shanghai, China).

### 4.4. RNA Preparation and RNA-seq Assay

Two single colonies of *C. albicans* wild-type strain were inoculated into 3 mL liquid YPD and incubated overnight at 30 °C on a 200 rpm shaker. The overnight cultures were diluted to OD600 of 0.1 in 10 mL YPD media and grown to OD600 of 0.6–0.8 at 30 °C with shaking. Then, the cells were treated with 0.015% MMS for 90 min before being harvested for RNA extraction. The cells with no MMS treatment were kept at 30 °C with shaking for 90 min and used as the control. Total RNA was extracted using Qiagen RNeasy plus Minikit [66]. RNA quality and quantity were checked using an Agilent Bioanalyzer. Sequencing was carried out at the Quebec Genome Innovation Center. *C. albicans* SC5314 haplotype A, version A22 downloaded from the Candida Genome Database (CGD) (http://www.candidagenome.org/, accessed on 8 June 2022), was used as a reference genome. Raw and processed data have been deposited in NCBI’s Gene Expression Omnibus (GSE196244, data set 1).

To confirm the RNA-seq data, an independent RNA-seq assay was carried out with similar treatments. Three single colonies of wild-type strain were inoculated and treated with 0.015% MMS as before. Total RNA was extracted using a trizol reagent kit (Invitrogen, Carlsbad, CA, USA) according to the manufacturer’s protocol. RNA quality was assessed on an Agilent 2100 Bioanalyzer (Agilent Technologies, Santa Clara, CA, USA) and checked using RNase-free agarose gel electrophoresis. After total RNA was extracted, eukaryotic mRNA was enriched by Oligo(dT) beads (Epicentre, Madison, WI, USA). Then, the enriched mRNA was fragmented into short fragments using fragmentation buffer and reverse transcribed into cDNA with random primers. Second-strand cDNA was synthesized by DNA polymerase I, RNase H, dNTP, and buffer. Then, the cDNA fragments were purified with a QiaQuick PCR extraction kit (Qiagen, Venlo, the Netherlands), end-repaired, A base added, and ligated to Illumina sequencing adapters. The ligation products were size selected using agarose gel electrophoresis, PCR amplified, and sequenced using Illumina Novaseq6000 by Gene Denovo Biotechnology Co. (Guangzhou, China). *C. albicans* SC5314 downloaded from NCBI (https://www.ncbi.nlm.nih.gov/genome/21?genome_assembly_id=294796, accessed on 8 June 2022) was used as a reference genome. Raw data have been deposited in the NCBI SRA database (PRJNA811694, data set 2).

The genes with consistent transcription (log2 fold change higher than 1) repeated in two independent assays were considered as defined MMS-responsive genes in this study (Table S3). In addition, the genes that showed altered transcription (log2 fold change higher than 1 and *p* value lower than 0.05) in only one RNA-seq assay were considered as putative MMS-responsive genes (Table S4).

### 4.5. Real Time PCR (qRT-PCR)

To confirm the transcription of specific genes, qRT-PCR was performed. The sample of the wild-type strain and the *RAD53* deletion strain were collected as before with or without treatment of 0.015% MMS for 90 min. Total RNA was extracted using a quick total RNA extraction kit (TR150, Tianmo Biotech, Beijing, China), and cDNA was synthesized using the qPCR RT Master Mix (TOYOBO, Shanghai, China) containing DNase for removing residual DNA in the template. The qRT-PCRs were performed using ChamQ SYBR qPCR Master Mix (Vazyme, Nanjing, China) according to the protocol from the manufacturer. Specific primers for target genes and the GAPDH primers used as control are listed in Table S8. The data for each gene was repeated at least three times.

### 4.6. Construction of the CAP1 Deletion Strain

To check the potential involvement of *CAP1* in MMS resistance, two alleles of the *CAP1* gene were substituted with the *HIS1*- or *ARG4*-containing repair cassettes successively in SN148 background. The repair DNA fragments were amplified from the pFA-HA-HIS1 plasmid or the pFA-HA-ARG4 plasmid with primers CAP1-re-F and CAP1-re-R [67]. The correct knockout strains were confirmed by PCR using primers CAP1-Te-F and CAP1-Te-R. Over two independent *CAP1* deletion strains were constructed, and two of them were used for phenotypic assays. 

### 4.7. Overexpression of GST2 and GST3 Genes in C. albicans

To increase the expression of *GST2*, the ORF of the *GST2* gene was amplified with primers GST2-OV-F and GST2-OV-R. The *ADH1* promoter was amplified with primers ADH1-F and ADH1-R from the genome of *C. albicans*, and cloned into the Ecor V and Ava I sites of the CIP10 plasmid [68], generating CIP10-ADH1p. The ORF of *GST2* was then cloned into the Xho I and Knp I sites of the CIP10-ADH1p plasmid, which contains an *ADH1* promoter, generating CIP10-ADH1p-GST2. Similarly, the ORF of *GST3* was cloned into the CIP10-ADH1p plasmid, generating CIP10-ADH1p-GST3. These two plasmids expressing *GST2* or *GST3* were then linearized by Stu I and transformed into wild-type BWP17 strain. Two independent transformants were tested in a phenotype assay, showing similar results. 

### 4.8. SOD Activity and GST Activity Assay 

The total SOD activity and glutathione S-transferase (GST) activity were measured using kits (A001-3A and A004-1-1) from Nanjing Jiancheng Bioengineering Institute (Nanjing, China). Total protein of *C. albicans* cells was obtained using the cell lysis buffer (P0013) from Beyotime (Shanghai, China), adding 10 mM phenylmethanesulfonyl fluoride (PMSF) as protease inhibitor. Protein concentrations were determined using a Bradford protein assay kit (GK5011) from Generway Biotech (Shanghai, China) [69]. The enzyme activity was measured according to the protocols supplied with the kits. For GST activity, the reaction was kept at 37 °C for 20 min, and the absorbance of GSH unbound from 1-chloro-2,4-dinitrobenzene (CDNB) was measured at OD405. For total SOD activity, the reaction was kept at 37 °C for 60 min and the absorbance was measured at OD450. The enzyme activity was normalized according to the total protein concentration, and the relative activity in the wild-type group was considered as 1.

### 4.9. Protein Extraction and Western Blot Assay

To detect the expression *RAD53*, a strain carrying an HA tag at the C terminal of Rad53 was used [33]. The log phase cells were treated with Congo Red or CFW for 90 min before harvesting. Protein extraction was performed as before, using a cell lysis buffer from Beyotime, China (P0013), adding 10mM phenylmethanesulfonyl fluoride (PMSF) as a protease inhibitor. Protein concentrations were determined using a Bradford protein assay kit from Generway Biotech, China (GK5011). Western blot analysis was carried out as previously described [33]. To detect the expression of Rad53-HA, a rabbit monoclonal anti-TAP antibody (Thermo Bioscience Inc. Madison, WI, USA) and the goat anti-rabbit IgG secondary antibody were purchased from Transgen, China.

### 4.10. Statistical Analysis

Statistical analysis was performed using GraphPad Prism 8.0.1. The significance between two groups was compared using a two-tailed paired *t* test. 

## Data Availability

The RNA-seq data have been deposited in NCBI’s Gene Expression Omnibus (GSE196244) and the NCBI SRA database (PRJNA811694).

## Supplementary Materials

The following supporting information can be downloaded at: https://www.mdpi.com/article/10.3390/ijms23147555/s1.
